# Starving Drug Resistance

**DOI:** 10.1097/HS9.0000000000000495

**Published:** 2020-11-10

**Authors:** Melania Tesio

**Affiliations:** Laboratory of Onco-hematology, Institut Necker Enfants Malades (INEM), Institut National de la Recherche Médicale (INSERM) U1151, Paris, France.

Metabolic rewiring is an important force shaping drug resistance in hematological malignancies. This aspect is particularly relevant in acute myeloid leukemia (AML), which is frequently characterized by the persistence of chemo-resistant leukemic stem cells (LSCs) accounting for disease relapse and poor prognosis. Hence, the development of more effective therapies for high risk AML patients relies, at least in part, on a better understanding of leukemia metabolic vulnerabilities and its translation into clinical settings.

Mitochondrial metabolism is central in the bioenergetics of AML cells. LSCs survival strictly depends on the mitochondrial oxidative phosphorylation (OxPHOS, ie, the progressive oxidation of nutrients to generate ATP synthesis),^1^ as well as on a protective uptake of functional mitochondria from bone marrow stromal cells.^2^ Noteworthy, the anti-apoptotic protein BCL-2 regulates OXPHOS in LSCs, thus supporting clinical trials combining the BCL-2 inhibitor venetoclax with hypomethylating agents (azacitidine or decitabine) for chemotherapy-ineligible AML patients.^3^ Elevated OXPHOS levels, moreover, drive resistance to the front-line induction drug cytarabine (AraC).^4^ In line with this, OXPHOS inhibitors sensitize AML cells to AraC in pre-clinical models.^4^ Nevertheless, despite venetoclax-based regimens showed good remission rates in chemotherapy-ineligible patients, they induced low response rates in individuals who did underwent treatment and were either refractory or relapsing.^5^ Furthermore, since mitochondria regulates important aspects of normal hematopoietic stem cells biology, caution has to be taken when inhibiting OXPHOS.

**Figure 1:**
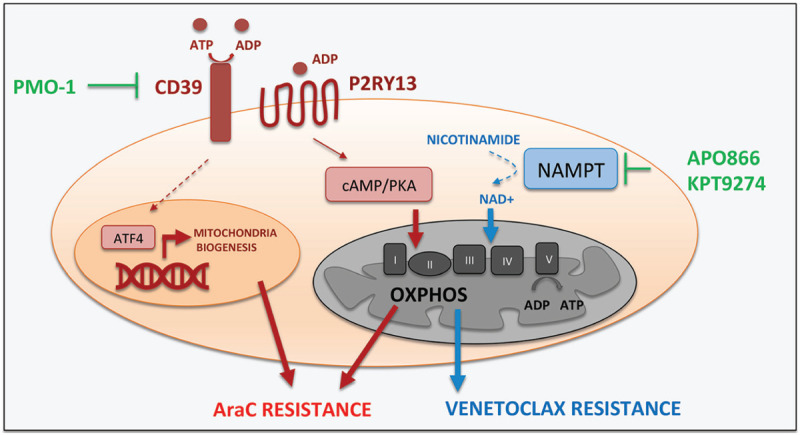
**CD39 and NAMPT are 2 druggable targets to selectively inhibit mitochondrial metabolism in drug resistant AML cells.** CD39, which drive resistance to AraC, promotes mitochondrial biogenesis via ATF4 and drives OXPHOS via a P2RY13-cAMP/PKA signaling. NAMPT, which mediates venetoclax resistance, drives OXPHOS by activating the NAD^+^-dependent catabolism of amino acids and fatty acids.

Facing these challenges, 2 recent papers identified novel and selective druggable targets to dampen OXPHOS and restore metabolic vulnerability in drug resistant AML cells. Building up on their previous works, the group of Craig Jordan investigated the low response rate induced by venetoclax-based regimens in relapsed/refractory AML patients.^6^ By comparing the metabolic profile of LSCs isolated from untreated patients or relapsed/refractory patients, the researchers demonstrated that relapsed/refractory LSCs have a unique metabolism which relies on nicotinamide. The authors demonstrated that LSCs metabolize nicotinamide into NAD^+^, which is an essential cofactor in enzymatic reactions occurring during the metabolism of aminoacids and the oxidation of fatty acids. As such, in relapsed/refractory LSCs, NAD^+^ enables the catabolism of aminoacids and fatty acids to drive OXPHOS and circumvent venetoclax-mediated cytotoxic effects (Fig. 1). Interestingly, the genetic and pharmacological inhibition of nicotinamide phosphoribosyltransferase (NAMPT), the rate-limiting enzyme in nicotinamide metabolism, eradicated LSCs in xenograft models. These effects, moreover, were selective as the low doses required to inhibit NAMPT in relapses/refractory LSCs allowed to spare normal hematopoietic stem/progenitor cells. Whereas more work will be necessary to translate these findings into clinical settings, these data are intriguing as they suggest a therapeutic window to target LSCs in high risk AML patients, currently disposing of limited therapeutic options.

A promising therapeutic target emerges also from the study conducted by Nesrine Aroua and colleagues, who investigated the mitochondrial-mediated resistance to AraC treatment.^7^ The researchers demonstrated that CD39, an enzyme which hydrolyses extracellular ATP into ADP, drives AML cells resistance to AraC by controlling mitochondria function at multiple levels. First, it promotes mitochondrial biogenesis by up-regulating the transcriptional factor ATF4. Second, it increases OXPHOS by eliciting an ADP-mediated activation of the purinergic receptor P2RY13 and its downstream PKA signaling (Fig. 1). In line with this, the genetic and pharmacological inhibition of CD39 enhanced AraC cytotoxicity in AML cells both in vitro and in vivo. These effects were selective as the CD39 inhibitor POM-1 did not enhance the sensitivity of normal human hematopoietic progenitor cells to AraC treatment.

In conclusion, the studies by Jones and Aroua present 3 major merits. First, they identified new selective targets to improve chemotherapy response in AML patients. Second, they demonstrate that these targets can be used as metabolic predictors of chemotherapy response. Indeed, NAMPT expression levels correlated with poor survival outcome. Similarly, high CD39 expression levels stratified high risk patients within the favorable cytogenetic risk subgroup. Last, but not least, they provide targets which can be blocked with compounds (such as the NAMPT inhibitor APO866 and anti-CD39 monoclonal antibodies), which are already undergoing testing in clinical trials.
